# Pathogenic Basis of Thromboinflammation and Endothelial Injury in COVID-19: Current Findings and Therapeutic Implications

**DOI:** 10.3390/ijms222112081

**Published:** 2021-11-08

**Authors:** Yasutomi Higashikuni, Wenhao Liu, Takumi Obana, Masataka Sata

**Affiliations:** 1 Department of Cardiovascular Medicine, The University of Tokyo, Tokyo 113-8655, Japan; wenhao-liu@g.ecc.u-tokyo.ac.jp (W.L.); obanatakumi@gmail.com (T.O.); 2 Department of Cardiovascular Medicine, The University of Tokushima, Tokushima 770-8503, Japan

**Keywords:** COVID-19, endothelial injury, inflammation, platelet activation, SARS-CoV-2, therapeutics, thrombosis

## Abstract

Coronavirus disease 2019 (COVID-19), caused by severe acute respiratory syndrome coronavirus 2 (SARS-CoV-2), has become a global pandemic with a great impact on social and economic activities, as well as public health. In most patients, the symptoms of COVID-19 are a high-grade fever and a dry cough, and spontaneously resolve within ten days. However, in severe cases, COVID-19 leads to atypical bilateral interstitial pneumonia, acute respiratory distress syndrome, and systemic thromboembolism, resulting in multiple organ failure with high mortality and morbidity. SARS-CoV-2 has immune evasion mechanisms, including inhibition of interferon signaling and suppression of T cell and B cell responses. SARS-CoV-2 infection directly and indirectly causes dysregulated immune responses, platelet hyperactivation, and endothelial dysfunction, which interact with each other and are exacerbated by cardiovascular risk factors. In this review, we summarize current knowledge on the pathogenic basis of thromboinflammation and endothelial injury in COVID-19. We highlight the distinct contributions of dysregulated immune responses, platelet hyperactivation, and endothelial dysfunction to the pathogenesis of COVID-19. In addition, we discuss potential therapeutic strategies targeting these mechanisms.

## 1. Introduction

Coronavirus disease 2019 (COVID-19) is an infectious disease caused by severe acute respiratory syndrome coronavirus 2 (SARS-CoV-2), which was originally isolated from the respiratory epithelium of patients with unexplained pneumonia in Wuhan, China, in late December 2019 [1,2,3]. Since the first report, COVID-19 has spread rapidly worldwide and has become a global pandemic with an unprecedented impact on social and economic activities. COVID-19 leads to atypical interstitial bilateral pneumonia and acute respiratory distress syndrome (ARDS) with high mortality in about 20% of infected patients [4], although for approximately 80% of infected patients, the symptoms of COVID-19 are similar to the common flu, including a high-grade fever and a dry cough, and spontaneously resolve within 6–10 days [5,6].

Thromboembolic complications are a major cause of morbidity and mortality in patients with COVID-19 [7]. The incidence of deep venous thrombosis and massive pulmonary embolism has been reported to be high with the rates of up to 86% and 36% in critically ill patients with COVID-19 who were admitted to the intensive care unit (ICU) due to severe respiratory distress [8,9,10,11,12,13,14,15,16,17]. A meta-analysis of three prospective and four retrospective studies demonstrated a significantly increased risk of pulmonary embolism and deep venous thrombosis among COVID-19 versus non-COVID-19 patients hospitalized in the ICU with the relative risk of 3.10, although this difference was not observed in non-ICU patients [18]. In addition, COVID-19 has been implicated in the incidence of arterial thrombotic diseases with a high burden of thrombi such as ischemia stroke, myocardial infarction, and acute limb ischemia [19,20,21,22,23]. One study reported the higher rate of revascularization failure due to recurrent thrombosis in the treatment of peripheral artery thrombosis manifesting as acute limb ischemia in COVID-19-related pneumonia patients compared with that in patients presenting with acute limb ischemia without COVID-19-related pneumonia during a similar period in 2019, although low oxygen pressure, rather than COVID-19-related pneumonia, was significantly associated with revascularization failure [24]. Postmortem studies have shown extensive microvascular thrombosis and occlusion in the lung of severe COVID-19 patients [25,26,27,28,29,30]. The incidence of alveolar capillary microthrombosis was nine times higher in COVID-19-related than in influenza virus-related respiratory failure patients [30]. Occluding microvascular thrombi have been observed not only in the lung, but also in other organs, including the heart, the liver, and the kidneys in critically ill COVID-19 patients [31]. These findings are distinct pathological features from fatal respiratory illness by infection of other coronarviruses, including SARS-CoV and Middle East respiratory syndrome coronavirus (MERS-CoV), which also caused global pandemics in 2003 and 2012, respectively [32,33,34]. Collectively, systemic thromboembolism, including venous thromboembolism, arterial thrombosis, and thrombotic microangiopathy, is a unique and essential feature of COVID-19. Of note, pre-existing cardiovascular risk factors, such as obesity, diabetes mellitus, hypertension and advanced age, are associated with high risk of thromboembolic events and death from COVID-19 [35,36,37,38].

Thromboinflammation is an emerging concept that refers to pathological responses within the vasculature due to blood vessel injury or infectious or noninfectious inflammation [39,40,41]. The pathological responses in thromboinflammation share common interacting detrimental processes, such as thrombus formation through activation of platelets and coagulation cascade and activation of the innate and adaptive immune systems. Thromboinflammation causes endothelial damage by producing proinflammatory cytokines and activating platelets and the complement system. This vicious cycle occurs in COVID-19 [5,7]. This review discusses the pathogenic basis of thromboinflammation and endothelial injury in COVID-19 and therapeutic implications.

## 2. Genome Composition, Structure, and Lifecycle of SARS-CoV-2

**Figure 1:**
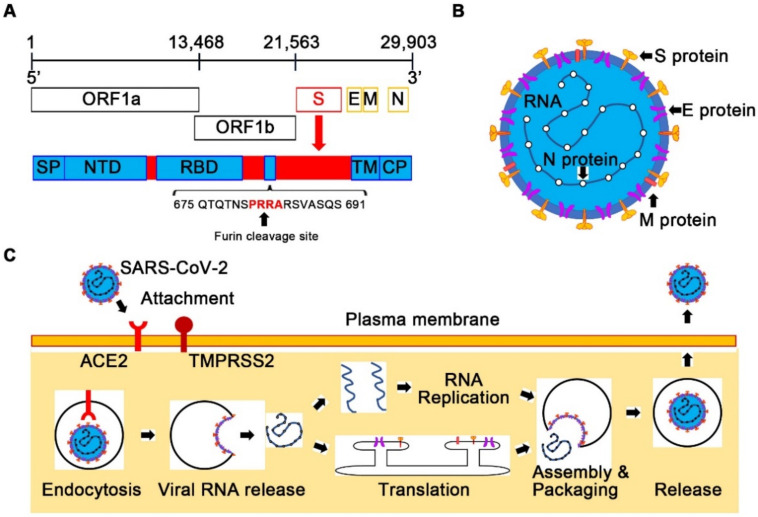
Genome composition, structure, and life-cycle of SARS-CoV-2. (**A**) Genome composition of SARS-CoV-2. The genome of SARS-CoV-2 is a positive-sense single-stranded RNA. Nonstructural proteins involved in genome transcription and replication are encoded in the open reading frame 1a (ORF1a) and ORF1b. The four ORFs encode the main structural proteins: the spike (S), envelope (E), membrane (M), and nucleocapsid (N) proteins. The S protein has the insertion of four amino acid residues (PRRA) that generate a polybasic cleavage site (RRAR). CP, cytoplasmic domain; NTD, N-terminal domain; RBD, receptor-binding domain; SP, signal peptide; TM, transmembrane domain. (**B**) Structure of SARS-CoV-2. SARS-CoV-2 virion consists of the envelope with three structural proteins, such as the S, E, M proteins, and the genomic RNA with the N proteins. (**C**) Life-cycle of SARS-CoV-2. SARS-CoV-2 enters host cells via its host cellular receptor, angiotensin-converting enzyme 2 (ACE2). The transmembrane protease serine 2 (TMPRSS2) cleaves and primes the receptor-bound S protein of SARS-CoV-2 for membrane fusion, which results in the release of the viral RNA genome into the cytoplasm. Translated viral structural proteins and replicated genomic RNA are assembled into the newly formed viral particles. Progeny viruses are released from the host cell by exocytosis.

SARS-CoV-2 belongs to the Betacoronavirus genus and shares 79% genome sequence identity with SARS-CoV and 50% with MERS-CoV [3,5,6]. The genome of SARS-CoV-2, which is a positive-sense single-stranded RNA, 29,903 bases in length, contains six functional open reading frames (ORFs) (Figure 1A). The ORF1a and ORF1b encode 16 nonstructural proteins (NSPs) involved in genome transcription and replication, including the RNA-dependent RNA polymerase complex, helicase, exonuclease, and proteases, similar to SARS-CoV. The other four ORFs encode the main structural proteins such as the spike (S), envelope (E), membrane (M), and nucleocapsid (N) proteins. In addition, several ORFs encoding accessory proteins with unknown functions are interspersed between the structural genes [42]. 

The SARS-CoV-2 virion has a diameter of approximately 100–200 nm, and the envelope consists of a lipid membrane and three structural proteins such as the S glycoprotein, the E protein, and the M protein [5,6] (Figure 1B). Within the viral envelope, the N protein holds the SARS-CoV-2 genomic RNA. SARS-CoV-2 has a distinct feature from other related viruses in the S protein [2,3,6]. The S protein contains the receptor-binding domain (RBD), which is an important protein for viral infection to host cells by binding to its receptor. The amino acid similarity between SARS-CoV-2 and SARS-CoV in this domain is only 73% [6]. The S protein of SARS-CoV-2 has the insertion of four amino acid residues (PRRA) at the junction of subunits S1 and S2, which generates a polybasic cleavage site (RRAR) [6,42,43] (Figure 1A). This amino acid sequence can be effectively cleaved by furin and other proteases. The furin cleavage site was reported to reduce the stability of the SARS-CoV-2 S protein, which might promote the binding of the S protein to its receptor through conformational adaptation, and thus, might explain the higher transmissibility of SARS-CoV-2 compared with SARS-CoV [44]. In addition to the S protein, the protein encoded by the ORF8 gene in SARS-CoV-2 is distinct from that in SARS-CoV with only 40% amino acid identity [6]. Unlike ORFs of SARS-CoV, ORF8 of SARS-CoV-2 does not contain a motif that triggers the intracellular stress pathway, which might explain the less deadly outcomes of COVID-19 compared with SARS [45].

When SARS-CoV-2 enters host cells, the RBD of the S protein binds to its host cellular receptor, angiotensin-converting enzyme 2 (ACE2) [6,43] (Figure 1C), which is also an attachment receptor for SARS-CoV, while MERS-CoV uses dipeptidyl peptidase 4 as the attachment receptor [34]. After binding to ACE2, the transmembrane protease serine 2 (TMPRSS2) cleaves and primes the receptor-bound S protein for fusion of the viral envelope and the target cell membrane. After translation of viral proteins and replication of the viral genome in the cytoplasm, progeny viruses are released from the host cell by exocytosis. Substantial expression of ACE2 and TMPRSS2 in nasal epithelial cells, lungs, and bronchial branches explains the tissue tropism of SARS-CoV-2 [6,46,47]. 

## 3. Dysregulated Immune Responses in COVID-19

Accumulating evidence suggests that a dysregulated immune response causes ARDS and thromboembolism in COVID-19 [48,49,50]. The innate and adaptive immune system contributes to the recognition and elimination of foreign pathogens in vertebrates. In critically ill COVID-19 patients, the innate immune system is hyperactivated, while the adaptive immune system is suppressed.

### 3.1. The Innate Immune System and COVID-19

**Figure 2:**
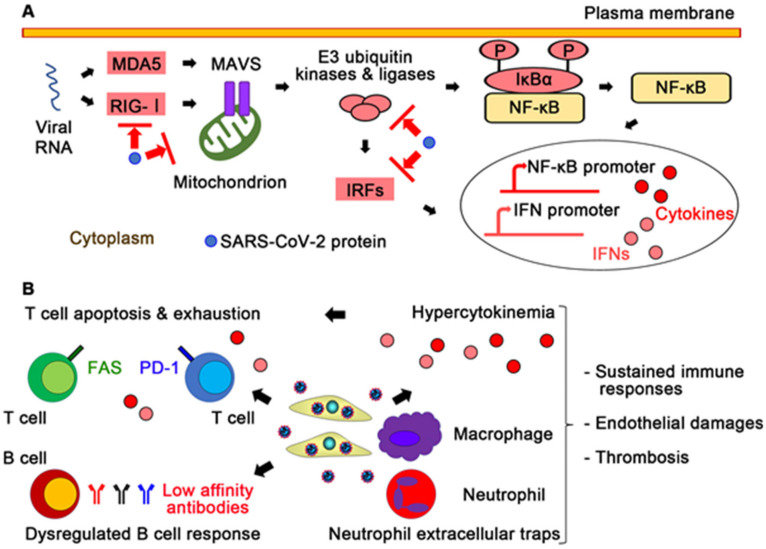
Dysregulated immune responses in COVID-19. (**A**) Suggested immune evasion mechanisms of SARS-CoV-2. SARS-CoV-2 proteins might inhibit pattern recognition receptors, such as retinoic acid-inducible gene I (RIG-I)-like receptors and melanoma differentiation-associated gene 5 (MDA5) and their downstream proinflammatory signaling. IFN, interferon; IRF, IFN-regulatory factor; MAVS, mitochondrial antiviral-signaling protein. (**B**) Hyperactivation of the innate immune system and suppression of the adaptive immune system in COVID-19. Persistent infection of SARS-CoV-2 induces hyperactivation of the innate immune system, subsequent hypercytokinemia, and the formation of neutrophil extracellular traps. Proinflammatory cytokines and activation-induced cell death signaling via programmed cell death protein 1 (PD-1) or the FAS receptor cause T cell apoptosis and exhaustion. While in COVID-19, B cells produce low affinity antibodies to SARS-CoV-2, which leads to further sustained SARS-CoV-2 infection. Dysregulation of the innate and adaptive immune systems results in sustained immune responses, endothelial damages, and thrombosis.

The innate immune system is the first-line defense system against viral infections [51]. The main components of the innate immune system are pattern recognition receptors (PRRs), proinflammatory cytokines, and immune cells such as macrophages and neutrophils. PRRs are expressed not only in immune cells but also in nonimmune cells [52] and recognize foreign pathogens on the surface of or within these cells. In infected cells, viral RNA activates PRRs, such as Toll-like receptors, retinoic acid-inducible gene I (RIG-I)-like receptors, and melanoma differentiation-associated gene 5 (MDA5), which leads to the activation of their downstream transcription factors, such as interferon regulatory factors (IRFs) and nuclear factor-κB (NF-κB) [53] (Figure 2A). These transcription factors induce the expression of type I and III interferons (IFN-I and IFN-III), proinflammatory cytokines and chemokines [34]. IFN signaling induces various proteins that contribute to reduction in protein synthesis, destruction of RNAs, and killing of infected cells. Immune cells modulate proinflammatory cytokine expression and removal of infected cells.

Like SARS-CoV and MERS-CoV, it has been suggested that SARS-CoV-2 has immune evasion mechanisms in which viral proteins suppress IFN production and IFN signaling [54,55,56,57,58] (Figure 2A). SARS-CoV-2 proteins inhibit signaling proteins, such as RIG-I, MDA5, mitochondrial antiviral-signaling protein (MAVS), E3 ubiquitin kinases and ligases, and IFN-regulatory factors (IRFs). On the other hand, NSP9 and NSP10 of SARS-CoV-2 might be able to promote proinflammatory cytokine expression through NF-κB signaling by inhibiting the NF-κB repressor NFRF [59]. In fact, in critically ill COVID-19 patients, IFN-I and INF-III levels and IFN-stimulated gene responses are lower compared with other respiratory viruses, while proinflammatory cytokines, including interleukin-2 (IL-2), IL-6, and tumor necrosis factor (TNF), are increased [60,61,62]. 

Single-cell RNA sequencing revealed an enrichment of SARS-CoV-2 RNA in the macrophage population from bronchoalveolar lavage samples of COVID-19 patients, which suggests SARS-CoV-2 directly infects macrophages [63]. In this study, higher chemokine expression was observed in the infected lung macrophages than in the uninfected lung macrophages [63]. Proteomic and metabolomic studies that compared the sera of severe COVID-19 patients with those of healthy individuals suggested that SARS-CoV-2 triggers macrophage polarization towards proinflammatory phenotype by downregulating important apolipoproteins for macrophage polarization towards anti-inflammatory phenotype [64]. In addition, neutrophil activation and the formation of neutrophil extracellular traps (NETs), which are pathogen-binding networks of extracellular fibers composed of DNA from neutrophils, have been implicated in disease severity in COVID-19 [65,66,67], although detailed mechanisms remain unknown. The formation of NETs in microvessels has been reported to be one of characteristic features in severe COVID-19 [68]. Collectively, SARS-CoV-2 has multiple mechanisms that activate and escape from the innate immune system. 

Proinflammatory cytokines and activated macrophages and neutrophils play important roles in activation of the coagulation system and inhibition of anticoagulant mechanisms [69] (Figure 2B). Proinflammatory cytokines, including IL-1β and TNF, induce expression of P-selectin, the von Willebrand factor (VWF), and fibrinogen to promote platelet binding in endothelial cells [70], whereas these cytokines suppress the protein C anticoagulant pathway by downregulating thrombomodulin and endothelial protein C receptor on endothelial cells [69]. Activated macrophages express a cell surface glycoprotein, tissue factor, to initiate the blood coagulation cascades and the generation of thrombin. Activated neutrophils produce a variety of toxic substances, including reactive oxygen species, and NETs, which enhance the coagulation pathways [66,70,71]. Collectively, hyperactivation of the innate immune system might contribute to thromboinflammation in severe COVID-19 patients. 

### 3.2. The Adaptive Immune System and COVID-19

The adaptive immune system is the pathogen-specific immune system that recognizes and removes a particular target. The main components of the adaptive immune system are T and B lymphocytes. CD8+ T cells, also known as cytotoxic T cells, kill virally infected cells, while CD4+ T cells, also called helper T cells, help activate macrophages and CD8+ T cells as well as B cells. Activated B cells differentiate into memory B cells, plasmablasts, or plasma cells that produce antibodies for activation of complement, opsonization to enhance phagocytosis, and neutralization.

It has been reported that the absolute numbers of CD4+ and CD8+ T cells are reduced in severe COVID-19 patients, which was also observed in SARS [72,73,74,75]. In addition to decreased T cell counts, reduced expression levels of T cell receptors subunits, T cell surface molecules, and their downstream signaling molecules were reported to be associated with COVID-19 severity [59]. Furthermore, CD8+ T cells from peripheral blood of COVID-19 patients, particularly those requiring ICU care, express high levels of exhaustion markers, including programmed cell death protein 1 (PD-1) and T cell immunoglobulin mucin-3 (TIM-3) [76]. Collectively, T lymphocyte function is globally impaired in severe COVID-19 patients, which might result in the failure to kill SARS-CoV-2-infected cells and sustained viral shedding and inflammation.

The mechanisms of suppressed T cell responses in severe COVID-19 remain largely unknown. Because ACE2 is not expressed in T cells, impaired T cell responses might not be due to direct toxic effect by SARS-CoV-2 [73]. It has been suggested that T cell lymphopenia might be caused by proinflammatory cytokines and activation-induced cell death [75,77] (Figure 2B). In fact, it is reported that T cells of patients with severe COVID-19 show high levels of apoptosis and increased expression of the death receptor FAS [77]. Interestingly, in SARS-CoV-2 infection, CD4+ T cells react to various viral proteins such as the S, N, and M proteins, whereas in SARS-CoV infection, most of CD4+ T cells dominantly recognize the S protein [78,79]. The lack of immunodominant CD4+ T cell response to SARS-CoV-2 might, in part, explain impaired T cell responses to SARS-CoV-2.

Dysregulated B cell response has been reported in COVID-19. Analyses of circulating B cells showed polyclonal expansion of plasmablasts and reduced memory B cells in severe COVID-19 patients compared with mild COVID-19 patients or healthy individuals [50,80,81] (Figure 2B). Although anti-SARS-CoV-2 antibodies are elevated in severe COVID-19 patients [82,83], their specificity and affinity seem to be low [84,85,86]. Thus, a robust and long-lasting protective humoral response might be impaired in SARS-CoV-2 infection, although the mechanism remains unclear.

Collectively, the adaptive immune system is dysregulated in severe COVID-19 patients. This might lead to sustained SARS-CoV-2 infection and innate immune responses, which might contribute to thromboinflammation in COVID-19.

## 4. Platelet Activation and the Coagulation Cascade in COVID-19

Thrombosis results from an imbalance between procoagulation and anticoagulation processes. Blood coagulation consists of two processes: platelet activation and fibrin formation. In response to endothelial damage, platelets bind to exposed collagen and VWF with platelet membrane glycoproteins for platelet adhesion and activation. Activated platelets release the contents of stored granules, which contain ADP, serotonin, platelet-activating factor, VWF, platelet factor 4, and thromboxane A2, for further activation. Activated platelets change their shape and membrane glycoprotein affinity to fibrinogen, which results in platelet aggregation to form the platelet plug. The coagulation factors drive the coagulation cascade through the contact activation pathway and the tissue factor pathway to form cross-linked fibrin for stabilization of the platelet plug. 

### 4.1. Platelet Dysfunction and COVID-19

**Figure 3:**
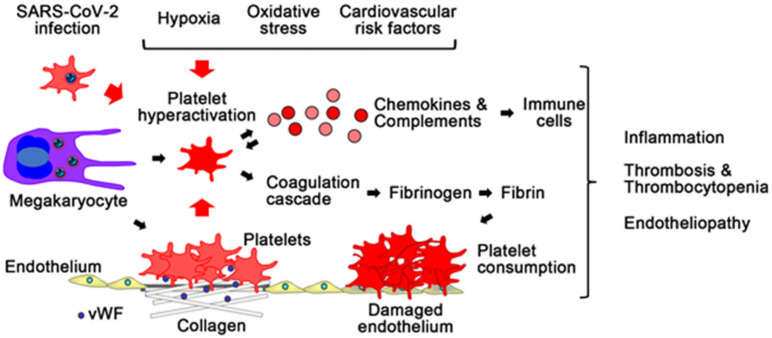
Platelet activation and the coagulation cascade in COVID-19. SARS-CoV-2 infection induces platelet hyperactivation. In COVID-19, platelets are primed to promote proinflammatory responses and activate the coagulation cascade and thrombosis. Hyperactivated platelets release large amounts of chemokines, and activate complements, which leads to increased fibrinogen, the von Willebrand factor (VWF), factor XII, and activation of immune cells. Platelet hyperactivation by SARS-CoV-2 infection causes inflammation, thrombosis with thrombocytopenia, and endotheliopathy. Hypoxia, oxidative stress, and cardiovascular risk factors might exacerbate these responses.

Platelet dysfunction has been implicated in SARS-CoV-2 infection [87,88,89] (Figure 3). Platelets form the platelet plug on injured endothelium through adhesion, activation, and aggregation. Activated platelets release various factors for activation of immune responses as well as promotion of the coagulation cascade, including calcium ion and coagulation factors [90,91,92]. In severe COVID-19 patients, a reduction in platelet count was reported to be as high as 35% [93]. On the other hand, in critically ill patients with COVID-19, bleeding events are rare, whereas thromboembolic complications are relatively high [94]. In addition, autopsy studies have reported the increased number of megakaryocytes, which produce platelets, in the organs, including the heart and the lungs [29,31]. Functional analysis of platelets demonstrated that platelets of COVID-19 patients release significantly larger amounts of cytokines, chemokines, and growth factors upon stimulation than platelets of healthy subjects, and contribute to increased fibrinogen, VWF, and factor XII, indicating that platelets in COVID-19 are primed to spread proinflammatory and procoagulant activities [88]. In addition, it was reported that the median activity of the VWF cleaving protease ADAMTS-13 (a disintegrin and metalloproteinase with a thrombospondin type 1 motif, member 13) in COVID-19 patients was lower than the expected median of the normal reference range, and the VWF to ADAMTS-13 ratio was associated with COVID-19 disease severity [95]. Interestingly, genetic analysis demonstrated that a missense variant of ADAMTS-13 was associated with ICU hospitalization in COVID-19 [96]. These findings suggested that SARS-CoV-2 infection is associated with consumption and hyperactivation of platelets.

It remains unclear whether SARS-CoV-2 directly affects platelet and megakaryocyte functions. Although it is controversial whether ACE2 is expressed in platelets and megakaryocytes sufficiently for SARS-CoV-2 entry [97,98,99,100], SARS-CoV-2 virions were detected by electron microscopy in megakaryocytes in the lungs of critically ill COVID-19 patients [31]. In addition, SARS-CoV-2 RNA or fragmented SARS-CoV-2 genome was found in platelets in some COVID-19 patients [97]. A recent report demonstrated SARS-CoV-2 internalization in platelets in vitro in both the presence and absence of the ACE2 inhibitor [100]. These findings suggest that megakaryocytes and platelets can take up SARS-CoV-2 independent of ACE2. In fact, influenza virus, which is a single-stranded RNA virus like SARS-CoV-2, can enter and activate platelet through PRRs such as TLRs [87,101]. In vitro experiments demonstrated that in platelets, incubation with SARS-CoV-2 leads to marked changes of the transcriptome for proinflammatory phenotype and up-regulation of programmed cell death markers [100,102]. Collectively, SARS-CoV-2 might directly activate platelets and lead to platelet apoptosis, proinflammatory cytokine release, and thrombus formation, which also induces endothelial damage by activating immune cells and the complement system.

Inflammation and various stressors, such as hypoxia and oxidative stress, can induce platelet activation [103,104]. Sustained immune responses to SARS-CoV-2 might lead to increased expression of cell surface proteins for platelet adhesion and activation. In addition, hypoxia and oxidative stress, which are induced by lung injury and immune responses in COVID-19, could affect platelet mitochondria metabolism and function to induce platelet activation and apoptosis [105]. In fact, a French study reported that a reduction in platelet count is associated with requirement of oxygen supplementation [93]. Collectively, SARS-CoV-2 infection might directly and indirectly induce platelet hyperactivation, which leads to thromboinflammation and endothelial injury in COVID-19. 

### 4.2. Coagulation Cascade and COVID-19

Coagulation abnormalities have been reported in COVID-19 patients. Elevated plasma levels of D-dimer, a fibrin degradation product, were often observed in COVID-19 patients, similarly to SARS-CoV-infected patients [7,15,37,72,106,107,108]. In addition, high D-dimer levels were correlated with a more severe disease course [106]. Many COVID-19 patients did not show an increase in prothrombin time (PT), assays evaluating the tissue factor pathway and common pathway of coagulation, although slightly prolonged PT was observed in nonsurviving COVID-19 patients. Furthermore, increased levels of fibrinogen, factor VIII, and VWF were observed in severely ill COVID-19 patients admitted to the ICU [15,109]. Together with mild, but not moderate-to-severe, thrombocytopenia, it is suggested that most COVID-19 patients have a hypercoagulable state, which is different from disseminated intravascular coagulation demonstrating elevated D-dimer levels, moderate-to-severe thrombocytopenia, prolonged PT, and decreased fibrinogen levels. Elevated levels of factor VIII and VWF might reflect the involvement of endothelial dysfunction in a hypercoagulable state because these factors are produced predominantly in endothelial cells [110]. 

Coagulation factors contribute to platelet activation, endothelial dysfunction, and inflammation [111]. Protease-activated receptors (PARs) play important roles in the mechanisms. PARs are G protein-coupled receptors that are activated by proteolytic cleavage of their extracellular domain. They are expressed in various types of cells, including platelets, endothelial cells, and immune cells. Thrombin activates PAR-1, -3, and -4, whereas factor VIIa and factor Xa cleave PAR-2. PAR-mediated signaling activates platelets for the release of their granules containing proinflammatory cytokines, morphological changes, and aggregation, as well as endothelial cells and leukocytes for production of chemokines and cytokines and upregulation of adhesion molecules. Collectively, the coagulation cascade might contribute to the vicious cycle of thromboinflammation and endothelial injury in COVID-19.

## 5. Endothelial Dysfunction in COVID-19

The endothelium maintains vascular integrity and barrier function. In addition, the endothelium regulates immune response and thrombus formation through production of several anti-inflammatory and antithrombotic factors, including nitric oxide, prostacyclin, thrombomodulin, activated protein C, tissue factor pathway inhibitor, and antithrombin, and through maintenance of glycocalyx [112,113,114,115]. Autopsy studies, using transmission electron microscopy, have demonstrated the presence of endothelial damage and apoptosis in blood vessels of COVID-19 patients [30,116]. Consistently, biomarkers of endothelial dysfunction, such as thrombomodulin, VWF, angiopoietin 2, and plasminogen activator inhibitor-1, are frequently elevated, and seem to be associated with disease severity in COVID-19 patients [117,118]. These findings indicate that SARS-CoV-2 causes endothelial dysfunction and barrier disruption, which leads to immune cell infiltration, and proinflammatory cytokine production, as well as thrombosis.

### 5.1. ACE2 and Endothelial Dysfunction in COVID-19

SARS-CoV-2 may impair vascular homeostasis by directly infecting vascular endothelial cells. ACE2, the cell-entry receptor for SARS-CoV-2, is reported to be expressed in vascular endothelial cells [30]. In addition, SARS-CoV-2 was detected in pulmonary endothelial cells by electron microscopy in critically ill patients with COVID-19 [119]. Furthermore, SARS-CoV-2 successfully infected engineered human blood vessel organoids and activated mature mouse aortic endothelial cells [120,121]. These findings support SARS-CoV-2 tropism for vascular endothelial cells. 

**Figure 4:**
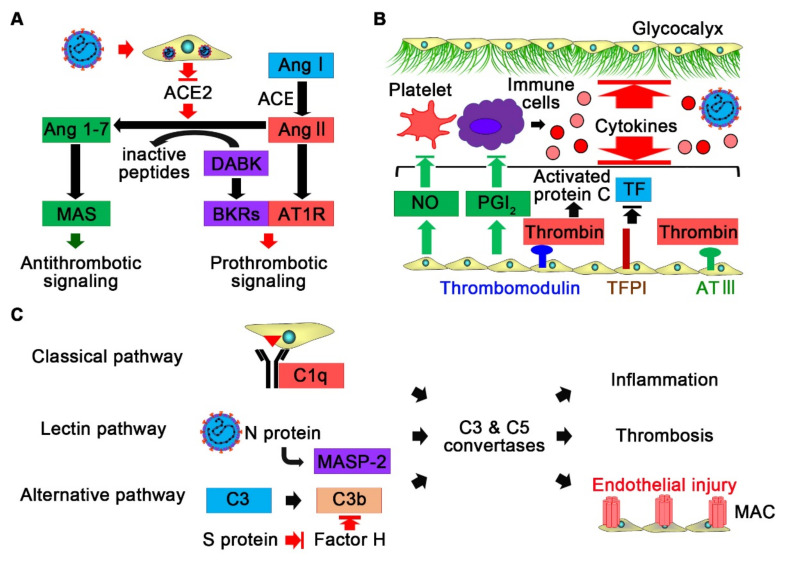
Endothelial dysfunction in COVID-19. (**A**) SARS-CoV-2 infection reduces ACE2 expression on endothelial cells, which leads to activation of the kallikrein–kinin system and the renin–angiotensin system. Increased des-Arg(9)-bradykinin (DABK) and angiotensin II (Ang II) activate bradykinin receptors (BKRs) and type 1 angiotensin receptors (AT1R) for prothrombotic signaling and endothelial dysfunction, whereas antithrombotic signaling via MAS receptors stimulated by Ang 1–7 is reduced. (**B**) Sustained immune responses in COVID-19 cause endothelial dysfunction through suppressing expression of anti-inflammatory and antithrombotic factors, such as nitric oxide (NO), prostacyclin (PGI2), thrombomodulin, activated protein C, tissue factor pathway inhibitor (TFPI), and antithrombin III (AT III), and degradation of glycocalyx. (**C**) SARS-CoV-2 infection activates the complement system via the classical, lectin, and alternative pathways. Activation of C3 and C5 convertases leads to inflammation, thrombosis, and endothelial injury. MAC, membrane attack complex; MASP-2, mannose-binding lectin-associated serine protease 2.

ACE2 is an enzyme attached to the membranes of cells that converts angiotensin II into angiotensin (1–7) (Figure 4A). Angiotensin (1–7) binds and activates the G protein-coupled receptor MAS, which counteracts to type 1 angiotensin receptor signaling by angiotensin II and exerts antioxidant, anti-inflammatory, and antithrombotic effects [122]. After binding by SARS-CoV-2, ACE2 is internalized, which leads to a reduction in ACE2 expression on endothelial cells [123]. SARS-CoV-2 S protein was reported to cause ACE2 destabilization with impaired mitochondrial function and endothelial nitric oxide synthase activity in endothelial cells [124]. Thus, SARS-CoV-2 infection to endothelial cells might result in angiotensin II hyperactivity to promote local proinflammatory and prothrombotic signaling. In addition, reduction in ACE2 expression by SARS-CoV-2 might activate the kallikrein–kinin system to increase vascular permeability through impairment in degradation of des-Arg(9)-bradykinin by ACE2 [70,125,126]. Prothrombotic signaling, such as the coagulation cascade, also leads to activation of the kallikrein–kinin system by producing coagulation factor XIIa, which activates prekallikrein. In critically ill COVID-19 patients, compared with healthy subjects, decreased prekallikrein and high molecular weight kininogen levels, as well as increased kallikrein–C1 inhibitor complexes, in plasma were reported, reflecting activation of the kallikrein–kinin system [127]. In addition, nearly all kallikreins are expressed in bronchoalveolar lavage fluid samples from COVID-19 patients but not detected in those from non-COVID-19 patients, including patients with bronchial asthma [125]. Collectively, SARS-CoV-2 might directly cause endothelial dysfunction and promote thrombus formation by inhibiting ACE2 on endothelial cells.

### 5.2. The Immune System and Endothelial Dysfunction in COVID-19

Sustained immune responses may also cause endothelial dysfunction and promote thrombosis in COVID-19 (Figure 4B). Proinflammatory cytokines induce expression of platelet binding-related proteins and suppress expression of anti-inflammatory and antithrombotic factors in endothelial cells [70]. TNF can degrade the endothelial glycocalyx by activating glucuronidases [70]. The endothelial glycocalyx inhibits coagulation and adhesion of immune cells and platelets by shielding the endothelial wall and mediating shear stress-induced nitric oxide release; thus, its disruption causes inflammation and thrombosis. 

In addition to proinflammatory cytokines, the complement system, which is a part of the immune system, may contribute to endothelial dysfunction and thrombus formation in COVID-19. The complement system is a cascade of serine proteases, and its activation results in stimulation of phagocytes and formation of the cell-killing membrane attack complex (MAC) [128]. The complement system can be activated by three biochemical pathways: the classical pathway, the alternative pathway, and the lectin pathway. The classical pathway is activated by binding of the complement component 1q (C1q) molecule to antigen–antibody complexes, while the alternative pathway is activated by spontaneous C3 hydrolysis, pathogens, or damaged cells. The lectin pathway is activated by binding of mannose-binding lectin (MBL) or ficolins to pathogens and subsequent activation of the MBL-associated serine protease 2 (MASP-2). In severe COVID-19 patients, components of the complement system have been reported to be upregulated compared with those in nonsevere COVID-19 patients, which was also observed in severe SARS and MERS patients [64,129,130,131]. 

Sustained immune responses and viral infection might activate the classical pathway and the alternative pathway (Figure 4C). The S proteins of SARS-CoV-2 were shown to directly activate the alternative pathway by interfering with the function of factor H, which accelerates decay of C3b [132]. In addition, it was reported that the N protein of SARS-CoV-2 can interact with MASP-2 to activate the lectin pathway [133,134,135]. Thus, SARS-CoV-2 may have several mechanisms to activate the complement system. MAC can induce endothelial cell injury and death. In addition, complement C5a can induce tissue factor expression in endothelial cells as well as platelet activation and aggregation. Furthermore, C5a induces NET formation through activation of its receptor on neutrophils, which triggers thromboinflammation and endothelial injury in COVID-19 [136]. Interestingly, genetic variants of complement pathway genes, including C3, factor H, and complement decay-accelerating factor, were reported to be significantly associated with clinical outcomes in COVID-19 [96,137]. Collectively, SARS-CoV-2 infection might result in unrestrained complement activation for endothelial cell dysfunction and thrombosis.

## 6. Cardiovascular Risk Factors and COVID-19

Pre-existing cardiovascular risk factors, such as obesity, diabetes mellitus, hypertension and advanced age, are associated with inflammation, platelet activation, and endothelial dysfunction, which play important roles in thromboembolism in COVID-19 [35,36,37,38]. In life style-related disease and aging, endogenous danger signals, including cell-free and mitochondrial DNA, heat shock proteins, and fatty acids, are released from various cells in response to mechanical and metabolic stresses, and activate PRRs to induce chronic inflammation, which recruits immune cells and impairs mitochondrial function and energy metabolism through expression of adhesion molecules and proinflammatory cytokines [52,138,139,140,141]. Mechanical and metabolic stresses also cause dysregulation of NAPDH oxidases and mitochondrial function, and induce oxidative stress [142,143,144]. In addition, age-related impairment in the nuclear factor erythroid 2-related factor 2 (NRF2) antioxidant response pathway leads to increased oxidative stress [145]. Increased oxidative stress can decrease the bioavailability of nitric oxide, which is a potent vasodilator and antiplatelet agent [138,146,147,148]. Chronic inflammation, increased oxidative stress, and decreased bioavailability of nitric oxide can induce platelet activation and endothelial dysfunction [138,149,150]. Collectively, SARS-CoV-2 infection and cardiovascular risk factors may synergistically exacerbate systemic inflammation, platelet activation, and endothelial dysfunction, which may explain the high risk of thromboembolic events in COVID-19 patients with these risk factors. 

ACE2, whose expression is reduced in COVID-19, is involved in cardiovascular diseases. ACE2-deficient mice have been reported to show cardiac contractile dysfunction and increased blood pressure [151,152]. Thus, SARS-CoV-2 infection might deteriorate cardiovascular diseases, including myocardial injury and heart failure [108]. 

ACE inhibitors and angiotensin II receptor blockers (ARBs) have been used for the treatment of cardiovascular diseases. In animal models, treatment with ACE inhibitors and ARBs have been shown to increase the expression of ACE2 [153,154], although it is controversial in clinical studies [155,156]. Increased expression of ACE2 might be harmful in terms of susceptibility to SARS-CoV-2 infection. On the other hand, ACE2 could be protective in cardiovascular disease through an increase in angiotensin (1–7). Two randomized clinical trials demonstrated that discontinuation of ACE inhibitors or ARBs did not affect the clinical outcomes of patients hospitalized with COVID-19 [157,158], whereas in one randomized control trial that enrolled both outpatients and hospitalized patients with COVID-19, discontinuing ACE inhibitors or ARBs was shown to lead to a faster and better recovery without affecting the maximum disease severity [159]. Since ACE inhibitors or ARBs are essential drugs for some patients with cardiovascular diseases, individualized therapeutic decision making might be important.

## 7. Therapeutic Approaches Targeting Thromboinflammation and Endothelial Injury in COVID-19

**Figure 5:**
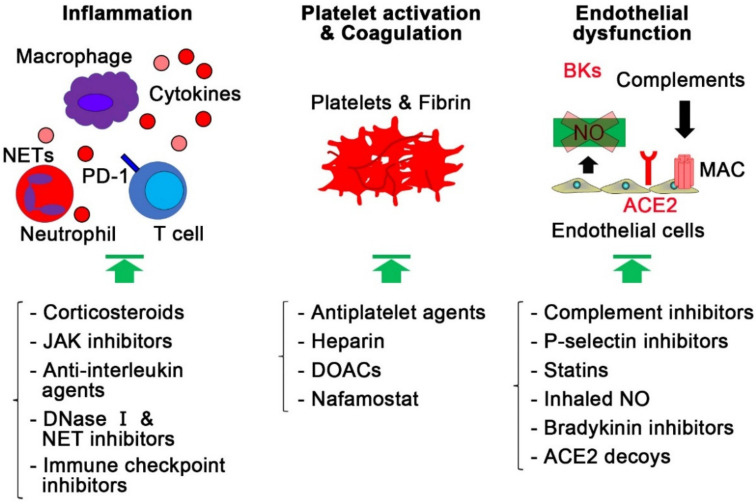
Therapeutic strategies targeting thromboinflammation and endothelial injury in COVID-19. In addition to vaccines or antiviral agents, therapeutic strategies, targeting inflammation, platelet hyperactivation, thrombosis, and endothelial dysfunction, might improve clinical outcomes, including mortality, in COVID-19 patients. ACE2, angiotensin-converting enzyme 2; BK, bradykinin; DOAC, direct oral anticoagulant; JAK, Janus kinase; MAC, membrane attack complex; NET, neutrophil extracellular trap; NO, nitric oxide; PD-1, programmed cell death protein 1.

Vaccines against SARS-CoV-2 have been developed and under distribution. Although these vaccines have been shown to be effective [160,161,162,163,164,165,166], eradication of COVID-19 is still challenging. In addition, antiviral agents, such as remdesivir and the combination of lopinavir and ritonavir, have been reported to be ineffective at improving mortality in COVID-19, although COVID-19 patients treated with remdesivir had a faster recovery time than those treated with placebo [167,168,169]. Therefore, it is important to discuss alternative therapeutic strategies targeting the pathological mechanisms of COVID-19, including inflammation, platelet activation, endothelial dysfunction, and thrombosis (Figure 5).

### 7.1. Targeting Dysregulated Immune Responses

Dysregulated immune responses play important roles in the pathogenesis of COVID-19. Corticosteroids are a class of steroid hormones that have anti-inflammatory effects by repressing proinflammatory mediators and inducing anti-inflammatory mediators [170]. A retrospective cohort study suggested that methylprednisolone treatment may reduce the risk of death by approximately 60 percent for COVID-19 patients with ARDS [171]. A quasi-experimental study reported that an early short course use of methylprednisolone improved the primary composite endpoint of death, ICU transfer, and mechanical ventilation with the adjusted odds ratio of 0.41 in moderately to severely ill patients with COVID-19 [172]. In a prospective meta-analysis, use of systemic corticosteroids was associated with lower 28-day all-cause mortality than usual care or placebo in critically ill patients with COVID-19 [173]. A large randomized controlled clinical trial showed that the use of dexamethasone for up to 10 days resulted in lower 28-day mortality than usual care with the relative risk reduction of approximately 35 percent or 20 percent in COVID-19 patients with invasive mechanical ventilation or oxygen support, whereas there was no benefit of dexamethasone use among COVID-19 patients without respiratory support [174]. Collectively, short-term use of corticosteroids at the early stage of moderate to severe COVID-19 might improve clinical outcomes, although use of corticosteroids has the potential risk of secondary infections, long-term complications, and prolonged SARS-CoV-2 infection.

Janus kinase (JAK) inhibitors are another type of immunomodulator that interfere with phosphorylation of signal transducer and activator of transcription proteins. By inhibiting intracellular proinflammatory signaling, JAK inhibitors reduce proinflammatory cytokine production. The ACTT-2 trial demonstrated that baricitinib, a JAK1 and JAK2 inhibitor, in combination with remdesivir reduced recovery time compared with remdesivir alone in COVID-19 patients receiving supplemental oxygen, but not invasive mechanical ventilation [175]. The COV-BARRIER trial reported that baricitinib in combination with dexamethasone reduced mortality in hospitalized COVID-19 patients who were not on invasive mechanical ventilation [176]. The STOP-COVID trial demonstrated that tofacitinib, another JAK inhibitor, had a survival benefit in hospitalized patients with COVID-19 pneumonia who were not on mechanical ventilation or extracorporeal membrane oxygenation [177]. Collectively, JAK inhibitors might be an option for the treatment of hospitalized COVID-19 patients who require high-flow oxygen and noninvasive ventilation.

More targeted therapies that inhibit proinflammatory cytokines have been considered for the treatment of COVID-19. Among cytokines, IL-6 and TNF-α levels at the time of hospitalization were reported to be independent predictors of disease severity and death in hospitalized patients with COVID-19 [178]. In a comparative study of SARS-CoV-2 infection with SARS-CoV and MERS-CoV that evaluated datasets from in vitro and in vivo experimental settings, SARS-CoV-2 has been shown to increase IL-6 levels to a larger extent than other viruses [179]. It was also reported that SARS-CoV-2 infection can induce IL-1β production through NLRP3 inflammasome activation, which might be associated with disease severity in COVID-19 [180]. Early studies suggested beneficial effects of the IL-6 inhibitor tocilizumab and the IL-1 receptor antagonist anakinra in severely ill COVID-19 patients [181,182,183]. In the two largest randomized controlled tocilizumab trials, the REMAP-CAP and RECOVERY trials, tocilizumab reduced mortality in severely ill COVID-19 patients requiring respiratory support associated with an inflammatory response [184,185]. Sarilumab, another IL-6 inhibitor, was similarly effective to tocilizumab in the REMAP-CAP trial [184]. The benefit of anakinra or a human monoclonal anti-IL-1β antibody, canakinumab, was inconclusive [186,187,188]. The timing for administration of anticytokine agents remains to be optimized. Collectively, IL-6 inhibitors could be an option for the treatment of severely ill patients with COVID-19. 

Extensive formation of NETs in microvessels is one of characteristic feature in COVID-19. Degradation of NETs by recombinant DNase I might be able to reduce inflammatory responses in COVID-19. Dornase alfa, a recombinant human DNase I, has been used, via inhalation, as a mucolytic agent to target NETs in cystic fibrosis patients. It was reported that endogenous DNase activity was reduced in severe COVID-19 patients compared with mild COVID-19 patients and healthy volunteers [189]. Several trials are under way to evaluate the efficacy of Dornase alfa in COVID-19. Dociparstat, a glycosamioglycan derivative of heparin, is another drug that may reduce neutrophil NET formation by inhibiting high mobility group box protein 1, platelet factor 4, and P-selectin. The efficacy of Dociparstat is under investigation in a randomized clinical trial [190].

Because the adaptive immune system is suppressed in severely ill COVID-19 patients, restoration of T cell counts and functions might be a potential therapeutic option. Supplementation of thymosin alpha I, which enhances cell-mediated immunity, has been reported to improve lymphocytopenia, T cell exhaustion, and clinical outcomes, such as mortality and need for invasive mechanical ventilation, in severe COVID-19 patients with respiratory distress [191]. Efficacy of immunotherapy targeting PD-1, which is an immune checkpoint and suppresses T cell inflammatory activity [192,193], is under investigation in ongoing clinical trials.

### 7.2. Targeting Platelet Hyperactivation and Coagulation Cascade

Platelet hyperactivation may contribute to sustained inflammatory responses and thromboembolism in the pathogenesis of COVID-19, which suggests that antiplatelet therapies might be effective for the treatment of COVID-19. A small clinical trial suggested that the use of dipyridamole might improve platelet counts and prevent the disease progression in COVID-19 patients [194]. A meta-analysis of five retrospective cohort studies with total of 14,065 participants demonstrated that COVID-19 patients treated with aspirin were associated with 53% decrease in mortality compared with those without aspirin [195]. However, randomized clinical trials reported that treatment with aspirin did not reduce mortality or disease progression in both inpatients and outpatients, although aspirin use slightly increased the rate of being discharged alive within 28 days in hospitalized COVID-19 patients [196,197]. Currently, more clinical trials evaluating the prophylactic use of aspirin and/or dipyridamole in COVID-19 patients are under way. Other anti-platelet agents, such as cilostazol, prostacyclin, and P2Y purinoceptor 12 inhibitors, might also be worth investigating for the treatment of COVID-19.

The coagulation cascade plays pivotal roles in endothelial dysfunction and thrombosis in COVID-19. Anticoagulants might be beneficial in the treatment of COVID-19. Randomized clinical trials demonstrated that therapeutic-dose anticoagulation with heparin did not improve clinical outcomes, including death, compared with usual-care thromboprophylaxis in severely ill COVID-19 patients admitted to the ICU [198,199]. However, one randomized clinical trial reported that therapeutic-dose of heparin increased the probability of survival to hospital discharge with reduced use of cardiovascular or respiratory organ support as compared with usual-care thromboprophylaxis in hospitalized COVID-19 patients without critical care-level organ support at enrollment [200]. In addition, another randomized trial demonstrated that therapeutic-dose low molecular weight heparin reduced major thromboembolism and death compared with standard heparin thromboprophylaxis among hospitalized non-ICU COVID-19 patients with very elevated D-dimer levels [199]. Collectively, routine use of heparin with therapeutic dose might not be beneficial in COVID-19 patients, although it could be one of therapeutic option in hospitalized patients with very high D-dimer levels.

Direct oral anticoagulants (DOACs), including factor Xa inhibitors, might suppress thromboinflammation and endothelial injury in COVID-19 patients through inhibition of PAR-mediated signaling. However, randomized clinical trials demonstrated that DOACs did not improve clinical outcomes in both inpatients and outpatients with COVID-19 [197]. In addition, rivaroxaban increased bleeding compared with prophylactic anticoagulation in hospitalized COVID-19 patients with elevated D-dimer levels. Although more clinical trials are under way, current findings would not support the use of DOACs in the treatment of COVID-19. The serine protease inhibitor nafamostat mesylate, which has short-acting anticoagulant effects and inhibits TMPRSS2 to suppress viral infection and replication [201], as well as factor Xa, factor XIIa, and factor VIIa, is under investigation in clinical trials.

### 7.3. Targeting Endothelial Dysfunction

Drugs that protect against endothelial dysfunction might be one of the therapeutic options. The complement system contributes to endothelial injuries, and several preliminary studies suggested favorable effects of C3 or C5a inhibition in severely ill patients with COVID-19 [202,203]. P-selectin on endothelial cells promotes thromboinflammation by promoting the adhesion of leukocytes and platelets, and thus, its inhibition might be beneficial to COVID-19 patients [204]. Inhibitors of the complement cascade, such as complement C5 inhibitors and P-selectin inhibitors, are under investigation in randomized clinical trials for the treatment of COVID-19 patients.

Statins have been reported to have pleiotropic and protective effects on endothelial cells [205]. A meta-analysis of 25 cohort studies involving 147,824 participants reported that the use of statin was associated with a lower risk of mortality in COVID-19 patients [206]. Randomized clinical trials are under way to further investigate the impact of statin use on clinical outcomes in COVID-19. Nitric oxide also inhibits thromboinflammation and reactive oxygen species production by acting on endothelial cells [207]. Inhaled nitric oxide could be a therapeutic option in COVID-19 [208]. Activation of the kallikrein–kinin system due to the down-regulation of ACE2 on endothelial cells might contribute to the pathophysiology of COVID-19, which suggests that inhibition of the kallikrein–kinin system might improve clinical outcomes of COVID-19 [209]. Icatibant, a bradykinin B2 receptor antagonist, is under investigation for the treatment of COVID-19. Since SARS-CoV-2 infects endothelial cells via ACE2, an ACE2 decoy might inhibit endothelial dysfunction by competitively binding to the SARS-CoV-2 spike RBD [210]. 

## 8. Discussion

Accumulating evidence demonstrates that SARS-CoV-2 infection directly and indirectly induces dysregulated immune responses, platelet activation, and endothelial dysfunction, which leads to a higher rate of thromboembolic events in severely ill patients with COVID-19 than in those without SARS-CoV-2 infection. Compared with severe SARS and MERS, characteristic clinical and pathological features of severe COVID-19 include milder thrombocytopenia, higher IL-6 levels, and more frequencies of extrapulmonary manifestations and diffuse microvascular thrombosis with NETosis [211,212,213]. Unlike SARS-CoV and MERS-CoV, it was reported that SARS-CoV-2 replicates to very high titer in the upper respiratory tract of patients in the presymptomatic phase, which leads to high transmissibility from human to human [211,214]. 

Based on the results of clinical trials, the current recommendation for the treatment of severely ill patients with COVID-19 receiving mechanical respiratory or circulatory supports is the use of dexamethasone. If these patients are within 24 h of admission to the ICU, dexamethasone plus tocilizumab, an IL-6 inhibitor, could be another option. For the treatment of hospitalized COVID-19 patients requiring oxygen delivery through a high-flow device or noninvasive ventilation, dexamethasone or dexamethasone plus remdesivir is recommended, while tocilizumab or baricitinib, a JAK inhibitor, can be added if rapidly worsening respiratory distress and systemic inflammation are observed in these patients. For hospitalized mild-to-moderate COVID-19 patients requiring supplemental oxygen, the use of dexamethasone and/or remdesivir is recommended. For COVID-19 inpatients without oxygen support or outpatients, there is insufficient evidence for therapeutic recommendation. Anticoagulation with prophylactic dose of heparin is recommended for hospitalized COVID-19 patients. Collectively, anti-inflammation drugs, especially targeting IL-6, is the main therapeutic option in addition to antiviral agents.

From mechanistic points of view, it remains unclear whether dysregulated immune response is the primary pathogenic mechanism in severe COVID-19, although in the acute phase of severely ill patients with COVID-19, anti-inflammation agents are effective. In a retrospective observational study, persistent endotheliopathy with increased levels of factor VIII, VWF, and thrombomodulin, compared with healthy subjects, was observed in convalescent COVID-19 patients, independent of ongoing acute phase response or NETosis [215,216]. The hypercoagulable state observed in severe COVID-19 patients was associated with elevated levels of factor VIII and VWF, suggesting the involvement of endothelial cells [110]. In addition, the elevated levels of VWF were also observed in mild COVID-19 patients [95]. Together with milder thrombocytopenia in severe COVID-19 compared with severe SARS and MERS, these findings suggest that endothelial dysfunction might be the main contributor to the pathogenesis of COVID-19. On the other hand, systemic endothelial dysfunction without detectable viral RNAs in the bloodstream or in the endothelium suggests important roles of thromboinflammation in the pathogenic mechanisms of COVID-19 [217]. The vicious cycle of interaction between thromboinflammation and endothelial injury might contribute to disease severity. Contribution of each pathogenic mechanism to COVID-19 might differ among patients, depending on their background characteristics and the stages of disease progression. In fact, pre-existing cardiovascular risk factors increase the incidence of thromboembolic events in COVID-19 [35,36,37,38].

Activation of the kallikrein–kinin system due to endothelial dysfunction has been implicated in the pathophysiology of COVID-19 [125]. ACE2 internalization and/or factor XII activation might be involved in this process. However, it remains unclear which of the two mechanisms mainly contribute to activation of the kallikrein–kinin system in COVID-19. It was reported that systemic factor XIIa levels were not elevated in patients with long COVID-19 syndrome, compared with healthy subjects, while factor VIII and VWF levels were higher in these patients, suggesting that ACE2 internalization might be the main contributor to activation of the kallikrein–kinin system in COVID-19 [215]. ACE2 decoys and bradykinin inhibitors could be therapeutic candidates for COVID-19 that target endothelial dysfunction, in addition to statin and nitric oxide.

In summary, therapeutic strategies targeting thromboinflammation and endothelial injury in COVID-19 have been in development by repurposing currently available drugs, in addition to vaccines or antiviral drugs [218]. Considering the multifactorial pathogenic nature of COVID-19, combinatorial treatment might be necessary to improve clinical outcomes, including mortality. Further studies that clarify the pathogenic basis of COVID-19 might provide new insights in determining the optimal timing and combinations of drug administration as well as creating novel therapeutic strategies.
